# Effects of hecogenin on Larvicidal activity against *Aedes aegypti *mosquito, the dengue vector

**DOI:** 10.1186/1753-6561-8-S4-P33

**Published:** 2014-10-01

**Authors:** Louise Oliveira, Débora Lacerda, Fabíola Nunes

**Affiliations:** 1 Biotechnology Center, Federal University of Paraiba, João Pessoa, Brasil

## Background

Dengue is a viral disease, caused by the dengue virus, a Flaviviridae family virus. Dengue is transmitted by several species of mosquito within the genus Aedes, principally A*. aegypti*. The dengue control is based on the mosquito combat, most of time through chemical insecticides. Hecogenin is a sapogenin present in the leaves of species from the Agave genus, with a wide spectrum of reported pharmacological activities. The present study was undertaken to evaluate the effect of hecogenin in *Aedes aegypti *mortality. Currently, several studies have shown the increase of the insect resistance for various chemical pesticides. In this way, the aim of this study was verify de larvicidal activity of the hecogenin acetate against A. *aegypti *larvae (L4).

## Methods

Twenty larvaes in the fourth stage (L4) were exposed to the concentration (20 mg/mL) of the hecogenin acetate for 5 days. The Hecogenin Acetate was dissolved in water and Tween 80. The control group consisted of 20 larvaes in the fourth stage, exposed to tap water plus tween 80 for 5 days.

## Results and conclusions

As results we found that the hecogenin acetate doesn't killed larvaes in the first 24 and 48 hours, killed 10% of larvaes after 72 hours, 80% of larvaes after 96 hours and 95% of larvaes in 120 hours in the concentration. This results confirm that the hecogenin have larvicidal activity against A. *aegypti*. As mechanism of action, it is possible that the acetate mimics the insect growth hormone, stopping its development and causing him to death.

